# RAC1 GTP-ase signals Wnt-beta-catenin pathway mediated integrin-directed metastasis-associated tumor cell phenotypes in triple negative breast cancers

**DOI:** 10.18632/oncotarget.13618

**Published:** 2016-11-25

**Authors:** Pradip De, Jennifer H. Carlson, Tyler Jepperson, Scooter Willis, Brian Leyland-Jones, Nandini Dey

**Affiliations:** ^1^ Department of Molecular & Experimental Medicine, Avera Research Institute, Sioux Falls, SD, USA; ^2^ Department of Internal Medicine, University of South Dakota SSOM, USD, Sioux Falls, SD, USA

**Keywords:** integrin-directed migration, RAC1 GTP-ase, brain-metastasis specific cells, actin cytoskeleton, Wnt pathway

## Abstract

The acquisition of integrin-directed metastasis-associated (ID-MA) phenotypes by Triple-Negative Breast Cancer (TNBC) cells is caused by an upregulation of the Wnt-beta-catenin pathway (WP). We reported that WP is one of the salient genetic features of TNBC. RAC-GTPases, small G-proteins which transduce signals from cell surface proteins including integrins, have been implicated in tumorigenesis and metastasis by their role in essential cellular functions like motility. The collective percentage of alteration(s) in *RAC1* in ER+ve BC was lower as compared to ER-ve BC (35% vs 57%) (brca/tcga/pub2015). High expression of RAC1 was associated with poor outcome for RFS with HR=1.48 [CI: 1.15-1.9] p=0.0019 in the Hungarian ER-veBC cohort. Here we examined how WP signals are transduced via RAC1 in the context of ID-MA phenotypes in TNBC. Using pharmacological agents (sulindac sulfide), genetic tools (beta-catenin siRNA), WP modulators (Wnt-C59, XAV939), RAC1 inhibitors (NSC23766, W56) and WP stimulations (LWnt3ACM, Wnt3A recombinant) in a panel of 6-7 TNBC cell lines, we studied fibronectin-directed (1) migration, (2) matrigel invasion, (3) RAC1 and Cdc42 activation, (4) actin dynamics (confocal microscopy) and (5) podia-parameters. An attenuation of WP, which (a) decreased cellular levels of beta-catenin, as well as its nuclear active-form, (b) decreased fibronectin-induced migration, (c) decreased invasion, (d) altered actin dynamics and (e) decreased podia-parameters was successful in blocking fibronectin-mediated RAC1/Cdc42 activity. Both Wnt-antagonists and RAC1 inhibitors blocked fibronectin-induced RAC1 activation and inhibited the fibronectin-induced ID-MA phenotypes following specific WP stimulation by LWnt3ACM as well as Wnt3A recombinant protein. To test a direct involvement of RAC1-activation in WP-mediated ID-MA phenotypes, we stimulated brain-metastasis specific MDA-MB231BR cells with LWnt3ACM. LWnt3ACM-stimulated fibronectin-directed migration was blocked by RAC1 inhibition in MDA-MB231BR cells. In the light of our previous report that WP upregulation causes ID-MA phenotypes in TNBC tumor cells, here we provide the first mechanism based evidence to demonstrate that WP upregulation signals ID-MA tumor cell phenotypes in a RAC1-GTPase dependent manner involving exchange-factors like TIAM1 and VAV2. Our study demonstrates for the first time that beta-catenin-RAC1 cascade signals integrin-directed metastasis-associated tumor cell phenotypes in TNBC.

## INTRODUCTION

Although a local disease at the initial stage, a pernicious progression of cancer begins as the disease starts to metastasize to the distant organs as a result of an invasion of tumor cells into surrounding/distant tissues [1]. Metastasis is one of the best-coordinated processes between tumor cells’ autonomous mechanisms and their microenvironment, and it comprises of several phenotypic steps including migration and invasion [2–4]. Metastasis requires tumor cell dissemination from the primary tumor to distant organs. Although metastasis occurs via both active and passive mechanisms, the initial steps in metastasis involve integrin-directed active migration of tumor cells following degradation of extracellular matrix (ECM) by matrix metalloproteinases [5]. Dissemination is a coordinated phenomenon of cell motility that requires the protrusion, chemotaxis, invasion and contractile activities of tumor cells to achieve integrin-directed (ID) cell migration [6]. Altered cell adhesion, increased cell migration, and invasion of tumor cells of a solid tumor are critical phenotypes during their spread to distant organs and thus are instrumental in the process of metastasis. Abnormal migration and invasion of tumor cells are key components of the metastatic phenotype [7] in solid tumors including breast cancers (BC) [8, 9]. These cells migrate in two distinct modes according to their cell type and their degrees of differentiation when invading the 3D environment, a rounded apolar ‘amoeboid’ movement or an elongated polar ‘mesenchymal’ movement [10] which involves cytoskeletal organization [11]. Anti-metastatic efficacy of several drugs in BC have been tested for their effectiveness in blocking tumor cell migration as well as invasion and potentially metastasis [7]. Thus, understanding the mechanism of regulation of migration has the potency to refine targeted therapies for treating cancers in a metastatic setting.

The interaction between metastasizing tumor cells and their ECM is known to be critical in mediating MA phenotypes including integrin-mediated migration, cytoskeletal organization, and morphological transitions [12, 13]. Among ECM components, integrins contribute to progression in many cancers, including breast cancers and down-regulation of surface alpha4beta1-receptor during oncogenic transformation critical for the establishment of the alpha5beta1-receptor-induced, MMP1–dependent invasive phenotype of breast cancer cells [14]. Integrins’ intracellular domains connect directly or indirectly to the actin cytoskeleton, thus linking the cytoskeleton to the ECM. Integrins constitute a highly dynamic and complex dialogue between the tumor epithelium and the surrounding microenvironment. The ECM–integrin axis initiates and maintains crucial cellular functions including survival and migration which are essential regulators of tumor cell phenotypes [15]. Integrins of the beta1 family and integrin-mediated signaling events have been reported to play an important role in breast tumor growth and progression [15, 16]. Thus, integrins contribute to cancer progression by mediating cancer cell invasion leading to metastasis [17]. Among integrins, fibronectin has been recognized as a crucial element in cell adhesion and migration. High fibronectin expression in breast tumor cells was associated with expression of MMPs including MMP7, a higher probability of metastasis, and poorer overall survival [18] revealing a direct relationship between integrin-directed migration and invasion of tumor cells [19, 20].

Rho GTPases contribute to different steps of cancer progression, including invasion and metastasis [21] and control signal transduction pathways linking cell surface receptors to a variety of intracellular responses including cell polarity and microtubule dynamics via the actin cytoskeleton [22, 23]. The signaling of the Rho family of small GTPases regulates the cytoskeleton-dependent processes during the cell migration. Cdc42 and RAC1 induce integrin-mediated cell motility and invasiveness [24]. RAC is a small GTPase of the Rho family that mediates stimulus-induced (including integrin) actin cytoskeletal reorganization to generate lamellipodia [25, 26]. RAC1 by its ability to (1) maintain cellular morphology, (2) maintain polarity and (3) to modulate actin cytoskeleton reorganization during an integrin-mediated directional movement of cells has been identified as the major mediator of metastatic progression. Activated RAC1 acts as an intracellular signal transducer to regulate multiple cellular events including cytoskeleton dynamics to maintain cellular morphology, polarity, adhesion and migration [27, 28]. By regulating cytoskeleton rearrangements, RAC1 GTPases play a vital role in MA cellular phenotypes including adhesion, migration, and invasion [29–31]. A key role for RAC1 in the regulation of cytoskeleton reorganization is to promote actin assembly required for the formation of lamellipodia and membrane ruffles [32]. Cancer cell motility occurs through actin reorganization and control of integrin-directed cell migration via the actin cytoskeleton providing the possibility of regulating cancer cell invasion and metastasis [33]. The “mesenchymal” movement (elongated movement of cancer cells) requires activation of RAC1 [34, 35], whereas rounded/amoeboid movement engages specific Cdc42 signaling pathways [36]. One of the ways RAC1 transduces signals is from integrins. RAC1 is involved in integrin-mediated adhesion [37]. Deregulated expression or activation patterns of RAC1 can result in aberrant cell signaling and numerous pathological conditions [38].

TNBC is an aggressive disease with poor clinical outcome [39, 40]. Although major advances have been achieved in the screening and treatment of primary tumors, management of metastasis still remain the leading cause of cancer-related death in TNBC. To metastasize to the host organ, tumor cell first dissociates from the primary tumor, traverses tumor basement membrane, and stroma, intravasate, survive in the circulation amongst the host of immune cells and finally, extravasate from the microvasculature to “home” in the secondary organ as it forms micro-metastasis. The intermittent steps involving integrin-directed migration and invasion have been described as *rate-limiting events* in metastasis in particular [41, 42]. Metastatic dissemination of the disease is the leading cause of TNBC associated mortality and currently, one-third of patients develops recurrence within three years of adjuvant therapy [43, 44]. In a highly aggressive and heterogeneous form of TNBC, tumor cells acquire key phenotypic characteristics typical for metastasis including integrin-directed aberrant migration and invasion through ECM following beta1 and beta4 integrin engagement [15]. Genetic alterations which cause deregulation of different signaling pathways are responsible for the acquisitions of these integrin-directed metastasis-associated (ID-MA) phenotypes which in turn determine the fate of the tumor cells. Our study demonstrated that an upregulation of the Wnt-beta-catenin pathway (WP) is one of the salient genetic features of TNBC and established that WP signaling in TNBC is associated with metastasis and poor prognosis [45]. We have also identified that the functional upregulation of secreted-MMP7, a transcriptional target of WP in TNBC is associated with the functional loss/absence of PTEN gene [46], the most common first event associated with basal-like subtype [47]. Thus, TNBC tumor cells can acquire ID-MA phenotypes which are imparted by WP alterations. The WP is a ligand-driven signaling pathway activation of which leads to a context-dependent transcription of beta-catenin target genes (http://www.stanford.edu/~rnusse/pathways/targets.html) that directly governs phenotypes including migration, polarity, and matrix remodeling [48] in several diseases including cancers [49]. Recently, we have identified the relevance of WP pathway in the biology of metastasizing TNBC tumor cells by undertaking a comprehensive study in which the involvement of WP was tested in the context of MA phenotypes and demonstrated that WP signals ID-MA tumor cell phenotypes in TNBC [50]. Since RAC1 activation instrumentally regulates the integrin-directed directional movement of tumor cells and WP activation in TNBC is functionally associated with ID-MA tumor cell phenotypes including migration and invasion, we hypothesized that WP regulates ID-MA tumor cell phenotypes of TNBC in RAC1-GTP-ase dependent manner.

Here we present evidence for the first time to demonstrate that the MA upregulation of WP signals for fibronectin-directed migration and invasion via activation of RAC1-GTPase and thus RAC1 activation acts as a downstream signal of WP activation in TNBC in the regulation of fibronectin-directed MA tumor cell phenotypes. The identification of the functional relationship between RAC1 signaling and the activation of WP in control of integrin-directed MA tumor cell phenotypes in TNBC mechanistically explain how the activation of WP in this subtype of BC is associated with the high metastatic incidences and a dismal outcome. Our study is the first report presenting that RAC1-activation via beta-catenin-VAV2/TIAM1 cascade acts as a downstream signaling event of WP activation in TNBC in the regulation of fibronectin-directed MA tumor cell phenotypes.

## RESULTS

### Alterations of *RAC1* gene in BC and different subtypes

**Figure 1:**
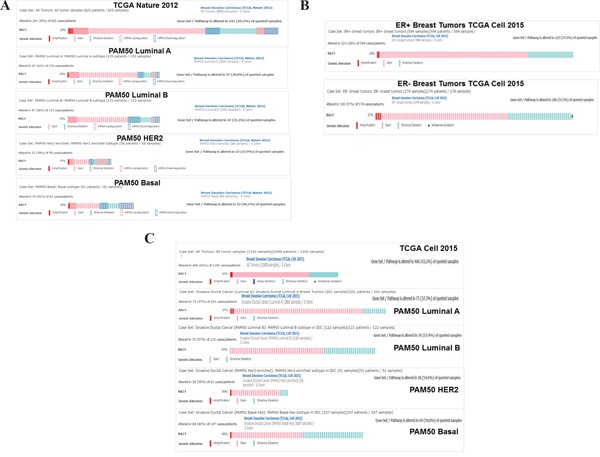
Alterations of RAC1 gene in breast cancers and breast cancer subtypes **A.** Oncoprints (cBioPortal) showing alterations in *RAC1* gene in Breast Invasive Carcinoma (TCGA Nature 2012) and subtypes including PAM50 Luminal A, PAM50 Luminal B, PAM50 HER2 and PAM50 Basal. The patient selected were, (1) Breast Invasive Carcinoma; (825 patients/825 samples), (2) Breast Invasive Carcinoma, PAM50 Luminal A (235 patients/235 samples), (3) Breast Invasive Carcinoma, PAM50 Luminal B (133 patients/133 samples), (4) Breast Invasive Carcinoma, PAM50 HER2 (58 patients/58 samples) and (5) Breast Invasive Carcinoma, PAM50 Basal (81 patients/81 samples). **B.** Oncoprints (cBioPortal) showing alterations in *RAC1* gene in ER+ve (upper panel) and ER-ve (lower panel) Breast Invasive Carcinoma (TCGA Cell 2015) brca/tcga/pub2015. The oncoprints are generated using 594 patients/594 samples for ER+ve and 174 patients/174 samples for ER-ve breast tumors. **C.** Oncoprints (cBioPortal) showing alterations in *RAC1* gene in Breast Invasive Carcinoma (TCGA Cell 2015) and subtypes including PAM50 Luminal A, PAM50 Luminal B, PAM50 HER2 and PAM50 Basal. The patient selected were, (1) Breast Invasive Carcinoma; (1105 patients/1105 samples), (2) Breast Invasive Carcinoma, PAM50 Luminal A (201 patients/201 samples), (3) Breast Invasive Carcinoma, PAM50 Luminal B (112 patients/112 samples), (4) Breast Invasive Carcinoma, PAM50 HER2 (51 patients/51 samples) and (5) Breast Invasive Carcinoma, PAM50 Basal (107 patients/107 samples). Advanced cancer genomic data visualization is obtained with the help of “The Onco Query Language (OQL)”. Oncoprints (different levels of zoom) have been generated using cBioPortal. Individual genes are represented as rows, and individual cases or patients are represented as columns. Protein level obtained from IHC staining (cBioPortal).

Oncoprints showed alterations (amplification, gain, shallow deletion, mRNA upregulation and mRNA downregulation) of *RAC1* gene in multiple subtypes of BC from two data sets, (1) TCGA, Nature, 2012 (*cBioPortal*) and (2) TCGA, Cell 2015; brca/tcga/pub2015 (*cBioPortal*) (Figure 1A-1C). Figure 1A shows that the percentages of *RAC1* gene alterations in a data set of TCGA, Nature 2012. The overall frequency of *RAC1* gene alterations for the entire sample set (TCGA, Nature, 2012) was 29%. Breaking down by molecular subtypes of BC, *RAC1* gene alterations are observed in 20% in PAM50 Luminal A, 35% in PAM50 Luminal B, 37% in PAM50 HER2 enriched and 40% in PAM50 Basal-like. In the data set of TCGA, Cell 2015, *RAC1* gene was altered more frequently in ER-ve cases (57%) as compared to ER+ve samples (38%). For the samples set of TCGA, Cell 2015, *RAC1* gene was altered in 42% total while it was altered 37% in PAM50 Luminal A, 57% in PAM50 Luminal B, 55% in PAM50 HER2 enriched and 60% in PAM50 basal-like. These changes indicate that there is a consistent trend of higher levels of alterations in *RAC1* gene in both ER-ve samples and PAM50 basal-like type of BC as compared to ER+ve samples and total samples of breast invasive carcinoma tumors respectively. Amplification and gain of *RAC1* gene occurred more than 50% of the time in all four categories of tumors tested (PAM50 Luminal A, PAM50 Luminal B, PAM50 HER2 and PAM50 Basal) in TCGA, Nature 2012 (cBioPortal) samples. The oncoprints showed that the major alterations of *RAC1* gene were constituted by the amplification/gain of the gene as compared to other changes (Figure 1). Interestingly, when the specific alterations of *RAC1* gene in the PAM50 Basal tumors were considered, we observed that the gain and amplification of the *RAC1* gene comprised 20% of the total alterations. The higher expressions of RAC1 mRNA were associated with gain and amplification of *RAC1* gene (Supplementary Figure S1).

### RAC1 inhibitor, NSC23766 inhibited fibronectin-induced RAC1 activation and fibronectin-induced migration in TNBC cells

Integrin signaling represents integrin-GTPase interaction in the context of cancer cell growth and behavior [51]. Fibronectin stimulation has been shown to activate RAC1 which also signals the cytoskeleton-dependent processes taking place during the cell migration in tumor cells [52].

**Figure 2:**
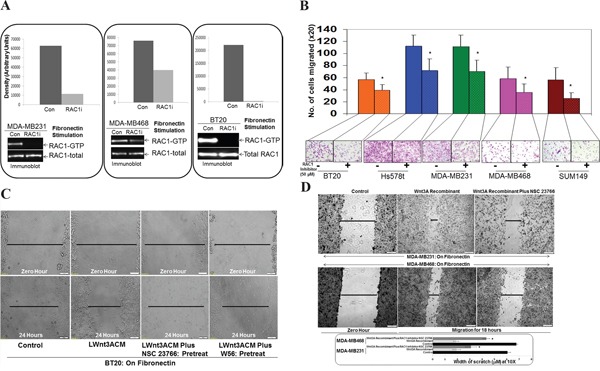
Effect of RAC1 Inhibitors, NSC23766 and W56 on fibronectin-induced RAC1 activation, fibronectin-induced migration, LWnt3ACM stimulated fibronectin-induced migration, and Wnt3A Recombinant stimulated fibronectin-induced migration in TNBC Cells **A.** NSC23766 inhibited fibronectin-induced RAC1 activation in MDA-MB231, MDA-MB468, and BT20 cells. TNBC cells were stimulated on fibronectin coated plates. RAC1 pull down was carried out on stimulated cells in the presence and absence of NSC23766. Total RAC1 were used as loading controls. Bar diagram shows changes in the relative density (Arbitrary Units). **B.** NSC23766 inhibited fibronectin-induced transwell migration in MDA-MB231, MDA-MB468, Hs578t, SUM149 and BT20 cells. Transwell plates were coated with fibronectin and migrated cells were counted following crystal violet staining. Bar diagram (*P< 0.05) shows a number of cells migrated on fibronectin (X20). **C.** NSC23766 and W56 blocked LWnt3ACM stimulated fibronectin-induced migration in BT20 cells. The scratch assay was performed on fibronectin-coated plates following pretreatment with RAC1 inhibitors. **D.** NSC23766 blocked Wnt3A Recombinant stimulated fibronectin-induced migration in MDA-MB231 and MDA-MB468 cells. Bar diagram (*P< 0.05) shows changes in the width of scratch (X10).

We used RAC1 Inhibitor, NSC23766 to test the involvement of RAC1 in fibronectin-induced RAC1 activation in MDA-MB231, MDA-MB468 and BT20 TNBC cells. In MDA-MB231, MDA-MB468 and BT20 TNBC cells, fibronectin stimulation caused an activation of RAC1 as demonstrated by the increased levels of RAC1-GTP. Although the degree of inhibition of RAC1 activation varied in between cell lines, inhibition of RAC1 by NSC23766 was observed in all cell lines tested (Figure 2A). Having confirmed the effect of the RAC1 inhibitor on the activation of RAC1 in cells, we tested the effect of the RAC1 inhibitor on fibronectin-induced migration in different TNBC cell lines. NSC23766 blocked fibronectin-induced transwell migration in MDA-MB231, MDA-MB468, Hs578t, SUM149 and BT20 TNBC Cells (Figure 2B). Migration of cells was found to be highest in Hs578t and MDA-MB231 cells. However, the inhibitory effect of NSC23766 was consistent with all cell lines as compared to their controls.

### RAC1 inhibitors, NSC23766 and W56 blocked both LWnt3ACM stimulated and Wnt3A Recombinant stimulated fibronectin-induced migration in TNBC cells

To verify that the effect of RAC1 inhibitor on the fibronectin-directed migration is WP specific, we used specific WP stimulation using both LWnt3ACM as well as Wnt3A Recombinant and tested the effect of two RAC1 inhibitors, NSC23766 and W56 on the LWnt3ACM stimulated and Wnt3A Recombinant stimulated fibronectin-induced migration in BT20, MDA-MB231 and MDA-MB468 TNBC Cells. Data showed that RAC1 inhibitors, NSC23766 and W56, blocked LWnt3ACM stimulated fibronectin-induced migration in BT20 TNBC cells (Figure 2C). Similarly, RAC1 Inhibitor, NSC23766 blocked Wnt3ARecombinant stimulated fibronectin-induced migration in MDA-MB231 and MDA-MB468 TNBC cells (Figure 2D).

### WP modulators, WntC59, and XAV939 blocked fibronectin-induced RAC1 activation, and Wnt3A Recombinant stimulated fibronectin-induced migration in TNBC cells

**Figure 3:**
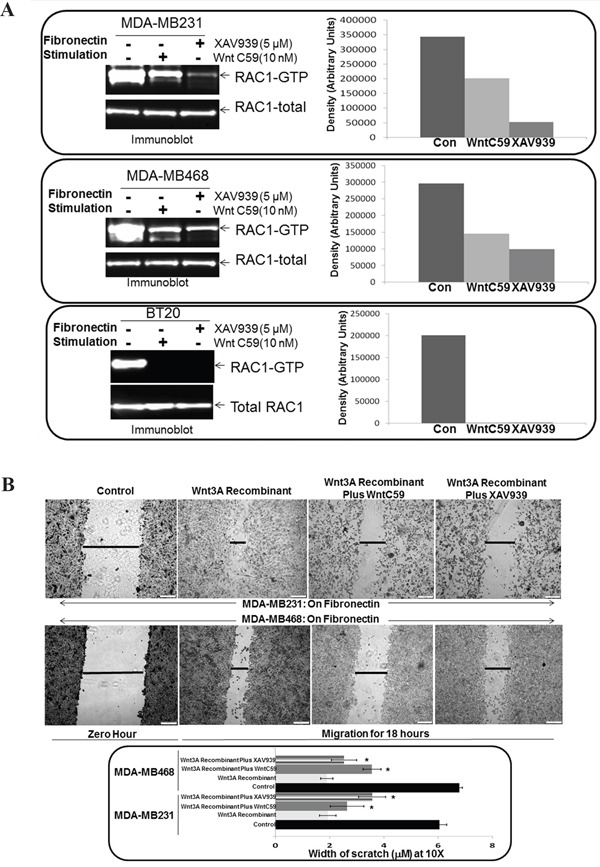
Effect of WP modulators, WntC59 and XAV939 on fibronectin-induced RAC1 activation and Wnt3A Recombinant stimulated fibronectin-induced migration in TNBC Cells **A.** WntC59 and XAV939 blocked fibronectin-induced RAC1 activation in BT20, MDA-MB231 and MDA-MB468 cells. Total RAC1 were used as loading controls. Bar diagram shows changes in the relative density (Arbitrary Units). **B.** WntC59 and XAV939 blocked Wnt3A Recombinant stimulated fibronectin-induced migration in MDA-MB231 and MDA-MB468 cells. Bar diagram (*P< 0.05) shows changes in the width of scratch (X10).

WP signals MA tumor cell phenotypes in TNBC. Earlier, we demonstrated that WP modulators, WntC59, and XAV939 blocked fibronectin-induced migration of TNBC cells [50]. To examine the role of WP in the activation of RAC1 in mediating fibronectin-induced migration in TNBC, we first tested fibronectin-induced RAC1 activation following WP modulators, WntC59, and XAV939. WntC59 and XAV939 blocked fibronectin-induced RAC1 activation in BT20, MDA-MB231 and MDA-MB468 TNBC cells (Figure 3A). Both inhibitors of WP signals blocked fibronectin-mediated RAC1 activation in different TNBC cells. We tested the specificity of the effect of WP inhibition by studying the effect of WntC59 and XAV939 on Wnt3A Recombinant stimulated fibronectin-induced migration of TNBC cells to show that WntC59 and XAV939 blocked Wnt3ARecombinant stimulated fibronectin-induced migration in MDA-MB231 and MDA-MB468 TNBC cells (Figure 3B).

### WP signaling inhibitor, sulindac sulfide decreased total beta-catenin as well as a transcriptionally active form of beta-catenin and blocked fibronectin-induced migration and matrigel invasion in TNBC cells

**Figure 4:**
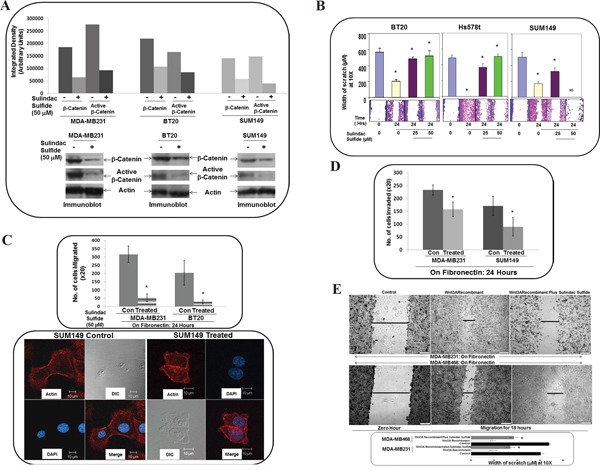
Effect of WP signaling inhibitor, sulindac sulfide on total beta-catenin and active beta-catenin, fibronectin-induced migration by scratch assay, fibronectin-induced transwell migration, matrigel invasion and Wnt3ARecombinant stimulated fibronectin-induced migration in TNBC cells **A.** Sulindac sulfide decreased total beta-catenin as well as transcriptionally active form of beta-catenin in SUM149, MDA-MB231 and BT20 cells. Cells were treated with sulindac sulfide and levels of total and active beta-catenin was determined by Western blots. Actin was used as loading controls. Bar diagram shows changes in the relative density (Arbitrary Units). **B.** Sulindac sulfide blocked fibronectin-induced migration in SUM149, Hs578t and BT20 cells. Bar diagram (*P< 0.05) shows changes in the width of scratch on fibronectin (X10). **C.** Sulindac sulfide blocked fibronectin-induced migration in SUM149, MDA-MB231 and BT20 cells. Bar diagram (*P< 0.05) shows a number of cells migrated on fibronectin across the membrane (X20) (upper panel). Migrated cells were stained with phalloidin 555 and counter stained with DAPI. The morphology of the migrated cells was compared with DIC images (lower panel). **D.** Sulindac sulfide blocked fibronectin-induced matrigel invasion in SUM149 and MDA-MB231 cells. Bar diagram (*P< 0.05) shows a number of cells invaded through matrigel across the membrane (X20). **E.** Sulindac sulfide blocked fibronectin-induced Wnt3ARecombinant stimulated migration in MDA-MB231 and MDA-MB468 cells. Bar diagram (*P< 0.05) shows changes in the width of scratch as the cell migrate on fibronectin (X10).

Beta-catenin is a functional as well as a biochemical readout of WP and changes in beta-catenin stability set the threshold of Wnt signaling [53]. We used WP signaling inhibitor, sulindac sulfide to downregulate cellular levels of beta-catenin in TNBC cell lines. Our *in vitro* phenotypic experiments focused on beta-catenin as it can be pharmacologically targeted (by sulindac sulfide) as well as tested in clinical trials [54, 55]. We used sulindac sulfide as a tool to test the role of RAC1 in mediating the effect of WP in ID-MA in TNBC. WP signaling inhibitor, sulindac sulfide decreased total beta-catenin as well as a transcriptionally active form of beta-catenin in SUM149, MDA-MB231 and BT20 cells (Figure 4A), a result we reported previously [50]. Sulindac sulfide blocked fibronectin-induced migration in transwell and scratch assay in SUM149, Hs578t, MDA-MB231 and BT20 cells (Figure 4B-4C) and abrogated fibronectin-induced matrigel invasion in SUM149 and MDA-MB231 cells (Figure 4D). The confocal microscopy of the stained migrated cells showed the disorganized morphology of cells following sulindac sulfide treatment (lower panel of Figure 4C).

### Sulindac sulfide blocked fibronectin-induced Wnt3ARecombinant stimulated migration in TNBC cells

To prove the specificity of the effect of sulindac sulfide in blocking WP mediated fibronectin-mediated migration, we tested the effect of sulindac sulfide on fibronectin-induced Wnt3ARecombinant stimulated migration in MDA-MB231 and MDA-MB468 cells. Figure 4E showed that sulindac sulfide abrogated fibronectin-induced Wnt3ARecombinant stimulated migration in TNBC cells.

### Actin cytoskeleton and podia-parameters of cells were altered following sulindac sulfide which abrogated fibronectin-induced activation of RAC1 in TNBC cells

**Figure 5:**
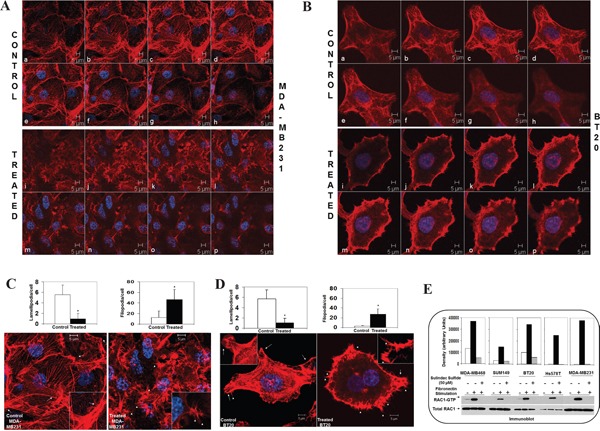
Effect of sulindac sulfide on fibronectin-induced cytoskeleton structure of filamentous actin, podia-parameters and activation of RAC1 and Cdc42 in TNBC cells **A-B.** Sulindac sulfide altered cytoskeleton structure of filamentous actin in MDA-MB231 and BT20 plated on fibronectin. **C-D**. Sulindac sulfide blocked lamellipodia while increased filopodia in MDA-MB231 and BT20 plated on fibronectin. Bar diagram (*P< 0.05) shows changes in the numbers of lamellipodia cell migrate on fibronectin. **E.** Sulindac sulfide abrogated fibronectin induced activation of RAC1 as determined from RAC1-GTP pull-down assay in MDA-MB231, MDA-MB468, BT20, Hs578t and SUM149 cells.

Having proved the WP specificity in the fibronectin-mediated migration in TNBC cells, we tested actin cytoskeleton structure and podia-parameters of cells following sulindac sulfide to show that sulindac sulfide altered actin cytoskeleton organization (Figure 5A-5B) and podia-parameters of MDA-MB231 and BT20 cells (Figure 5C-5D). Cells which failed to move on fibronectin following treatment with sulindac sulfide showed a significant reduction in the number of lamellipodia although filipodia formations were increased. We observed a similar effect of sulindac sulfide in different TNBC cells previously [50]. Finally, we tested whether sulindac sulfide treatment under similar condition had any effect on the activation of RAC1 following integrin engagement. Sulindac sulfide abrogated fibronectin-induced activation of RAC1 in MDA-MB231, MDA-MB468, BT20, Hs578t and SUM149 cells (Figure 5E). Although fibronectin stimulation activated RAC1 to a different degree in different TNBC cells, treatment with sulindac sulfide blocked the RAC1 activation in all TNBC cells.

### SiRNA mediated downregulation of beta-catenin protein blocked fibronectin-induced migration and fibronectin-induced matrigel invasion in TNBC cells

**Figure 6:**
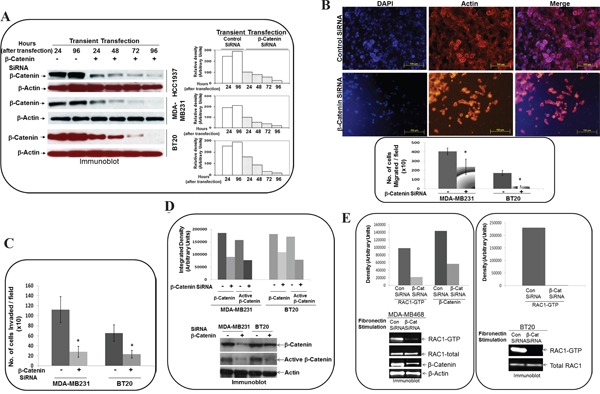
Effect of siRNA-mediated downregulation of beta-catenin on fibronectin-directed migration, matrigel-invasion, transcriptionally active beta-catenin and fibronectin-induced activation of RAC1 in TNBC cells **A.** BT20, MDA-MB231 and HCC1937 cells were transiently transfected with beta-catenin siRNA for 96 hours. Total levels of beta-catenin were determined by Western blots and compared between siRNA (24, 48, 72 and 96 hours) and control (24, and 96 hours) cells at similar time points. Actin was used as loading controls. Bar diagram shows changes in the relative density (Arbitrary Units). **B.** MDA-MB231 and BT20 cells were transiently transfected with beta-catenin siRNA for 24 hours and then allowed to migrate on fibronectin in a transwell assay. Migrated cells were stained with phalloidin 555 and DAPI using the cut-outs of transwell membrane for the picture (upper panel). Bar diagram (*P< 0.05) shows a number of cells migrated through transwell membrane (X10). **C.** MDA-MB231 and BT20 cells were transiently transfected with beta-catenin siRNA for 24 hours and then allowed to invade across matrigel on fibronectin. Bar diagram (*P< 0.05) shows a number of cells invaded across matrigel (X10). **D.** Active beta-catenin was determined from lysates of MDA-MB231 and BT20 cells which were transiently transfected with beta-catenin siRNA for 24 hours. Beta-catenin levels were used as the reference for the transient transfection of siRNA. Actin was used as loading control. Bar diagram shows changes in the relative density (Arbitrary Units). **E.** Active RAC1 (GTP-RAC1) and active beta-catenin were determined from lysates of MDA-MB468, which were transiently transfected with beta-catenin siRNA for 24 hours. Active RAC1 (GTP-RAC1) was determined from lysates of MDA-MB468, which were transiently transfected with beta-catenin siRNA for 24 hours. Actin and total RAC1 were used as loading controls. Bar diagram shows changes in the relative density (Arbitrary Units).

It has been established [56] that sulindac sulfide is a nonsteroidal anti-inflammatory drug or Cox inhibitor, affecting a wide array of a signaling pathway in addition to WP. Thus, we also used beta-catenin siRNA to verify the specificity of the effect. Transient transfection of beta-catenin siRNA (Figure 6A) at different time points starting from 24 hours to 96 hours caused downregulation of beta-catenin protein in HCC1937, MDA-MB231, and BT20 cells. Two different time points were included for the control siRNA. The effect of siRNA was observed from 24 hours and continued till 96 hours in all cell lines. SiRNA mediated downregulation of beta-catenin protein blocked fibronectin-induced migration in MDA-MB231 and BT20 cells (Figure 6B) as shown in the upper panel pictures of the stained migrated cells (DAPI, F-actin, and merge) and lower panel bar-diagrams made from the number of migrated cells. SiRNA mediated downregulation of beta-catenin protein blocked fibronectin-induced matrigel invasion in MDA-MB231 and BT20 cells (Figure 6C).

### SiRNA mediated downregulation of beta-catenin protein decreased transcriptionally active form of beta-catenin and blocked fibronectin-induced RAC1 activation in TNBC cells

To test the biochemical effect of beta-catenin siRNA on the WP, we tested the effect of siRNA-mediated downregulation of beta-catenin protein on the transcriptionally active form of beta-catenin in cells. As expected, siRNA mediated downregulation of total beta-catenin protein decreased transcriptionally active form of beta-catenin in MDA-MB231 and BT20 cells (Figure 6D) as previously reported by us in other TNBC cell lines [50]. Having proved the effect of beta-catenin siRNA on the WP signals, we set the stage to test the effect of downregulation of WP on the RAC1 activation in TNBC cells. SiRNA mediated downregulation of beta-catenin protein blocked fibronectin-induced RAC1 activation in MDA-MB468 and BT20 cells (Figure 6E).

### Fibronectin-induced activation of Cdc42 was abrogated by the RAC1 inhibitor, WP modulators, sulindac sulfide and transient transfection of beta-catenin siRNA in TNBC cells

**Figure 7:**
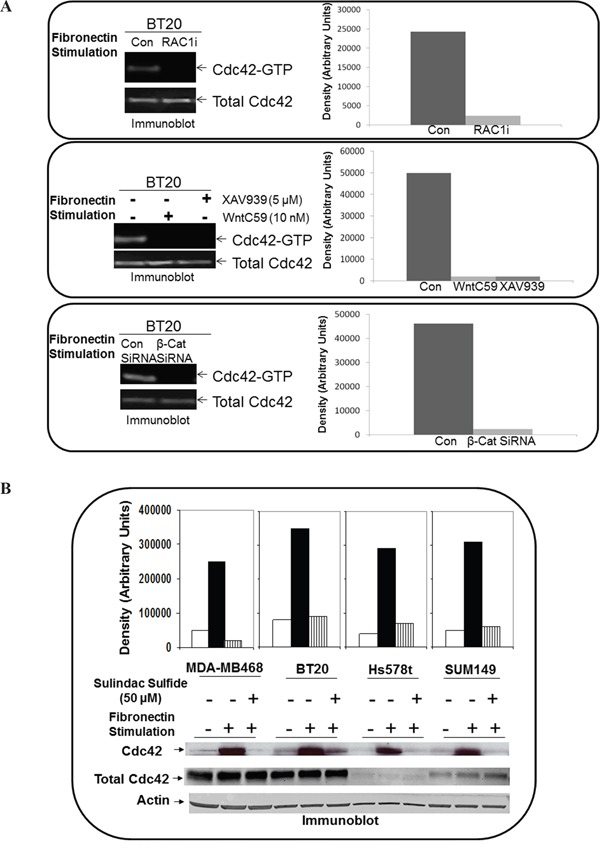
Effect of RAC1 inhibitor, WP modulators, sulindac sulfide and beta-catenin siRNA on fibronectin-mediated activation of Cdc42 in TNBC cells **A.** Active Cdc42 was pulled down from BT20 cells treated with NSC 23766 (upper panel), WntC59 and XAV939 (middle panel) and from BT20 cells transiently transfected with beta-catenin siRNA (lower panel). Total Cdc42 were used as loading controls. Bar diagram shows changes in the relative density (Arbitrary Units). **B.** Active Cdc42 was pulled down from MDA-MB468, BT20, Hs578t and SUM149 cells treated with sulindac sulfide. Actin and total Cdc42 were used as loading controls. Bar diagram shows changes in the relative density (Arbitrary Units).

Figure 7A shows that the fibronectin-induced activation of Cdc42 was blocked following RAC1 inhibitor, WntC59, XAV939 as well as transient transfection of beta-catenin siRNA in BT20 cells. Similarly, fibronectin-induced activation of Cdc42 was abrogated by sulindac sulfide in MDA-MB468, Hs578t, SUM149 and BT20 cells (Figure 7B).

### RAC1 inhibitor blocked fibronectin-induced LWnt3A conditioned media stimulated migration of brain metastasis-specific MDA-MB231BR TNBC cells

**Figure 8:**
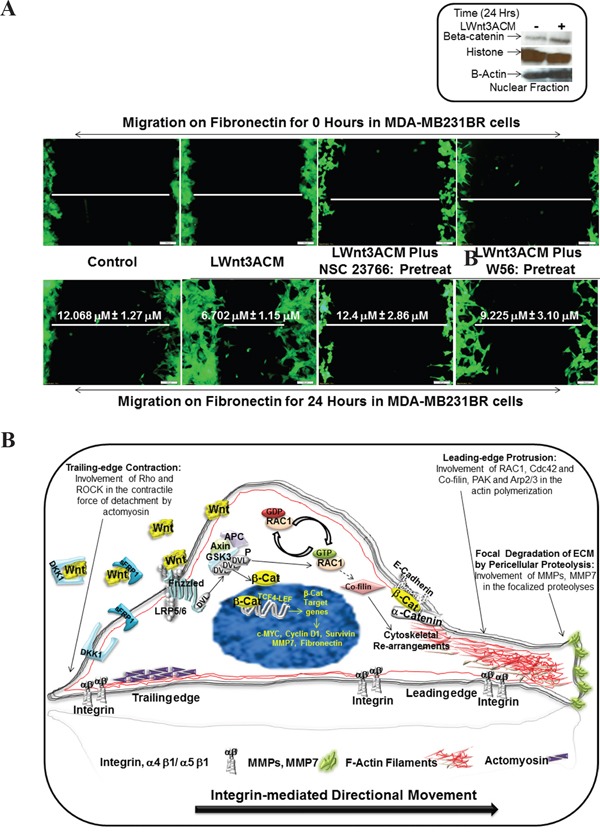
Effect of RAC1 inhibitors, NSC 23766 and W56 on LWnt3A-stimulated fibronectin-mediated migration of brain metastasis-specific TNBC cells **A.** EGFP-tagged brain metastasis-specific TNBC cells, MDA-MB231BR were allowed to migrate on fibronectin for 24 hours under LWnt3A stimulation in the presence or absence of RAC1 inhibitors. Zero hour control for each scratch was presented as the internal control. Activation of the canonical Wnt signaling pathway in MDA-MB231BR cells under LWnt3A stimulation (Inset). Wnt signaling is stimulated in TNBC cells with LWnt3ACM (+) at 24 hours, and is assessed based on the level of beta-catenin accumulation compared to the non-stimulated(-)-condition. Histones were used as a nuclear marker. Beta-actin expression was used as total protein loading control. **B.** Schematic representation of the mode of involvement of WP via activation of RAC1 in the integrin-mediating directional movement of tumor cells through actin cytoskeletal organization.

To test a direct cause-effect relationship between RAC1 and ID-MA phenotypes in TNBC and varify the specificity of WP in MA phenotype in TNBC, we tested the effect of RAC1 inhibitor on fibronectin-induced LWnt3A conditioned media stimulated migration of brain metastasis-specific MDA-MB231BR TNBC cells. NSC23766, a small molecule RAC1 inhibitor has been shown to inhibit RAC1 specifically without any activity against RhoGTPases [57]. Figure 8A showed that NSC23766 blocked fibronectin-induced LWnt3A conditioned media stimulated migration of brain metastasis-specific MDA-MB231BR TNBC cells. We performed an experiment demonstrating the activation of canonical WP signals following the exposure of cells to LWnt3ACM by testing the nuclear transportation of beta-catenin by nuclear fractionation. WB from the nuclear fraction showed that nuclear beta-catenin was increased in MDA-MB231BR cells 24 hours after LWnt3ACM stimulation. Data shows that Wnt signaling is stimulated in cells with LWnt3ACM (+) at 24 hours as evident from the increased levels of beta-catenin accumulation compared to the non-stimulated(-)-condition (Figure 8A, Inset). Figure 8B represents a proposed diagrammatic role of RAC1 activation in the integrin-mediated directional movement of TNBC cells under WP activation.

### Alterations of *TIAM1* and *VAV2* genes, exchange factors responsible for RAC1 activity in TNBC

**Figure 9:**
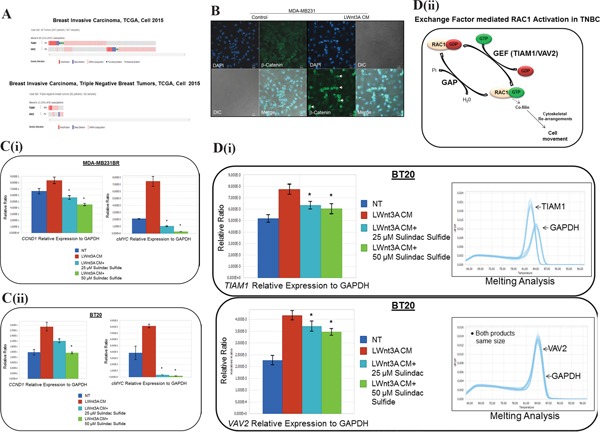
Mechanism of RAC1 activation in TNBC following WP stimulation **A.** Alterations of *TIAM1 and VAV2* genes (exchange factors for RAC1 activation) in breast invasive carcinoma and TNBC subtype:Oncoprints (cBioPortal) showing alterations in *TIAM1 and VAV2* genes in Breast Invasive Carcinoma (TCGA, Cell 2015; brca/tcga/pub2015) (upper panel) and TNBC subtype (lower panel). The patient selected were, (1) Breast Invasive Carcinoma; (817 patients/817 samples) and (2) Breast Invasive Carcinoma, TNBC (82 patients/82 samples). **B.** Activation and nuclear localization of beta-catenin following LWnt3A CM stimulation in MDA-MB231 cells. The White arrow indicates the nuclear localization of beta-catenin (green). DAPI was used for the nuclear staining. The merged picture indicates beta-catenin positive cells. DIC images were captured to test the condition of the cells. **C.** MDA-MB231BR (i) and BT20 (ii) TNBC cellswere stimulated with LWnt3A CM in the presence and absence of two doses of sulindac sulfide. The mRNA expression of beta-catenin target genes, *CCND1* and *cMYC* were quantified by qRT-PCR. **D.** Quantification of *TIAM1* and *VAV2* mRNA by qRT-PCR following sulindac sulfide treatment (25 μM and 50 μM) in BT20 cells under the stimulation of WP by LWnt3A condition media (LWnt3A CM). BT20 (i) TNBC cellswere stimulated with LWnt3A CM in the presence and absence of two doses of sulindac sulfide. The mRNA expression of genes for RAC1 exchange factors, *TIAM1* and *VAV2* were quantified by qRT-PCR. Melting curves were included in each case. Amplified genomic materials were analyzed for consistency via melting analysis. Each peak represents individual gene products according to base pair size. *VAV2* peaks overlap with *GAPDH* as they are nearly identical in amplicon size. The relative mRNA expression of *TIAM1* and *VAV2* genes to *GAPDH* (used as the reference) were determined using LightCycler96 software from Roche. Bars indicate mean ± SE (In triplicates). *P> 0.05. The exchange-factor mediated RAC1 activation in TNBC is schematically represented (ii).

To define the mechanism of activation of RAC1 in TNBC, we studied the alterations of *TIAM1* and *VAV2* genes in TCGA data set. Oncoprints showed alterations (amplification, deep deletion, mRNA upregulation truncating mutation and missense mutation) of *TIAM1 and VAV2* genes in invasive breast carcinomas and TNBC data sets, (Figure 9A). Figure 9A shows that *TIAM1 and VAV2* genes were altered in 6% of breast invasive carcinomas (upper panel). *TIAM1* and *VAV2* genes were altered in 10% and 7% respectively in TNBC subtype (lower panel).

### Nuclear localization of beta-catenin in TNBC cells following LWnt3A CM stimulation

We tested the nuclear localization of beta-catenin in TNBC cells following LWnt3A CM stimulation. The merged images of Figure 9B showed that following LWnt3A CM stimulation, nuclear localization of beta-catenin was increased in the cells.

### Transcriptional upregulation of beta-catenin target genes, *CCND1* and *cMYC* following LWnt3A CM stimulation

Quantification of beta-catenin target genes, *CCND1* and *cMYC* in MDA-MB231 and BT20 cells were carried out to test the activation status of WP. Figure 9C (i and ii) showed that both mRNAs for both target genes were significantly increased following LWnt3A CM stimulation, and this increase was blocked by the sulindac sulfide treatment. Figure 9C shows that the increase in the nuclear beta-catenin (Figure 9B) is capable of increasing the expression of mRNA (by qRT-PCR) levels for “transcriptional target genes of beta-catenin” like cyclin D1 and cMYC in MDA-MB231BR as well as in BT20 TNBC cells, and sulindac sulfide can block this increase. Both Figure 9B and 9C established the fact that in our model system, WP stimulation is functional.

### Mechanism of RAC1 activation via exchange factors, TIAM1 and VAV2 in TNBC

Once the status of WP stimulation/function was confirmed in our TNBC model system, we tried to seek the mechanism of RAC1 activation. Figure 9Di showed that mRNAs of RAC1 exchange factors *TIAM1* and *VAV2* were significantly increased following the LWnt3ACM stimulation in BT20 cells. The increase was blocked following 24 hours of the treatment with sulindac sulfide.

### Kaplan-Meier survival for RFS split on the median expression of Affymetrix probe 208640_at RAC1 in IHC determined ER-ve samples in the Hungarian Academy of Sciences (HAS) breast cancer cohort

**Figure 10:**
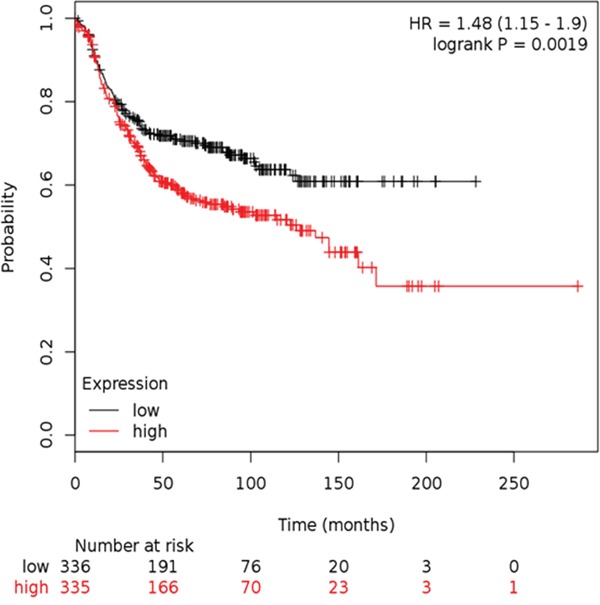
Kaplan-Meier survival for RFS split on the median expression of Affymetrix probe 208640_at RAC1 in IHC determined ER-ve samples in the HAS breast cancer cohort High expression of RAC1 denotes poor outcome.

Having demonstrated the mechanistic role of RAC1 activation in WP mediated ID-MA phenotypes in TNBC, we sought to define the relationship between RAC1 and outcome in ER-ve breast cancers. In the Hungarian Academy of Science breast cancer cohort, we observed that Kaplan-Meier survival for RFS split on the median expression of Affymetrix probe 208640_at RAC1 in IHC determined ER-ve samples. The mRNA expression of RAC1 in the Hungarian Academy of Science breast cancer cohort (http://www.ncbi.nlm.nih.gov/pubmed/20020197) ER-ve breast cancer cohort suggests that high expression denotes poor outcome for RFS HR=1.48 [CI:1.15-1.9] p=0.0019 when split on the median. High expression of RAC1 denoted poor outcome (Figure 10).

## DISCUSSION

Our study was built on two of our previous reports which established an association between WP and TNBC in the context of metastasis [45, 46] in line with others [58, 59]. Since TNBC is an aggressive disease, we then hypothesized that the upregulation of WP in this subtype has a functional relationship with ID-MA phenotypes which led to our third report demonstrating that WP signals ID-MA tumor cell phenotypes [50]. As migration occurs following an alteration of the adhesive property of cells and migration is required for tumor cell invasion and eventually metastasis, we tested the involvement of RAC1 downstream of WP pathway activation in the integrin-directed migration of TNBC cells. To determine the mechanism of WP action in the biology of ID-MA tumor cell phenotypes in this study, we used attenuators of WP signals including WntC59 and XAV939, beta-catenin siRNA, sulindac sulfide as well as stimulators of WP signals including Wnt3A recombinant protein and LWnt3ACM. Our study included TNBC cell lines representing all major types of genetic alterations as reported earlier [50]. Our validation strategy included alteration of beta-catenin because beta-catenin is the functional readout of WP. Hence, we pharmacologically (by sulindac sulfide) and genetically (siRNA) attenuated beta-catenin and studied the effect of this maneuver on ID-MA vis-à-vis activation of RAC1 and Cdc42. Two RAC1 inhibitors were used as internal controls to test the effect of RAC1 signals on the ID-MA phenotypes in TNBC. The specificity of the effect of WP attenuators was tested using them following the WP stimulators in the context of ID-MA phenotypes. The specificity of RAC1 activation in WP mediated ID-MA phenotypes in the context of metastasis were tested using brain metastasis-specific MDA-MB231BR TNBC cells.

The initial evidence for Rho GTPases in breast tumor invasion and metastasis *in vivo* came from studies expressing dominant negative forms of RhoA, RAC1 and Cdc42 [60]. It should be understood that the upregulation of RAC1 and Cdc42 signals occurs in other subtypes of BC. In fact, an increased migratory capacity of T47D ER+ve cells overexpressing Cdc42 and RAC has shown a disorganized morphology on collagen gels [24, 61]. We observed a similar morphological disorganization in TNBC cells those migrated poorly on fibronectin following sulindac sulfide (lower panel of Figure 4C) which resulted in abrogation of fibronectin-stimulated activation of RAC1 and Cdc42. A similar perturbation of fibronectin-stimulated activation of RAC1 and Cdc42 following the blockade of WP after WntC59, XAV939 treatment and transient transfection of beta-catenin siRNA was demonstrated in TNBC cells (Figure 3A, Figure 6E, Figure 7A) which were comparable to the inhibition of fibronectin-stimulated activation of RAC1 following RAC1 inhibitor (Figure 2A). RAC1 and Cdc42 regulate signal transduction pathways that mediate distinct cytoskeletal rearrangements required for the production of actin-rich protrusions called lamellipodia and filopodia and then subsequent cell migration [62]. Cell spreading of MTLn3 rat mammary adenocarcinoma cells was selectively abrogated by dominant negative N17RAC1 [60]. We have observed that perturbation of WP following sulindac sulfide treatment disorganized the cytoarchitecture of filamentous actin in TNBC cells (Figure 5A-5B). Our observation was in line with the loss of lamellipodia formation on fibronectin in the same TNBC cells following sulindac sulfide (Figure 5C-5D). We demonstrated that RAC1 inhibitors abrogated LWnt3ACM stimulated fibronectin-mediated migration of metastasis-specific TNBC cell lines (Figure 8A). Our study demonstrated for the first time that the RAC1 activation and fibronectin-mediated MA phenotypes are functions of WP in TNBC.

Increased protein levels of RAC and Cdc42 [63–65] have been reported in BC. Similarly the alterations of RAC1 gene are the highest in PAM50 Basal tumors (TCGA Nature2012) (40%), ER-ve breast tumors (Cell 2015) (57%) as well as PAM50 Basal tumors (TCGA Cell 2015) (60%), as compared to other BC subtypes (Figure 1). In the PAM50 Basal tumors, the combined percentage of the gain and amplification of *RAC1* gene was 20%, and the higher expressions of RAC1 mRNA were associated with gain and amplification of *RAC1* gene (Supplementary Figure S1). In the metastatic setting, an increased RAC1 GTP-ase protein has been observed in 100% of lymph node metastases [65]. Immunohistochemical staining of RAC1 showed weak RAC1 expression in benign breast disease but high expression levels in DCIS, primary breast cancer, and lymph node metastases. Breast tumor cells from patients with recurrent disease had RAC1 expression localized at the plasma membrane, suggesting activation of RAC1, in patients with aggressive BC [65]. Because GTP-bound RAC1 is translocated to the cell membrane, results suggest that constitutive activation of RAC1 signaling may be characteristically present in more aggressive forms of BC [61]. A direct involvement of the alpha5beta1 integrin (a component of fibronectin) in the regulation of actin-mediated migration and invasion via RAC1 has been reported. A cytosolic protein, Nischarin, binding the alpha5beta1 integrin (a component of fibronectin) plays an important role in migration by regulating the actin cytoskeleton. Nischarin inhibits RAC induced migration and invasion of BC cells by affecting signaling cascades involving PAK, an immediate downstream of RAC1 [66, 67]. We have observed that fibronectin stimulation causes activation of RAC1 and Cdc42 in five different TNBC cells (Figure 5E), and this activation was abrogated by an RAC1 inhibitor (Figure 2A).

We studied the signal(s) that initiated RAC1 activation in BC. Wild-type RAC GTP-ases cycle between active GTP-bound and inactive GDP-bound states and the conversions are mediated by Guanine nucleotide Exchange Factors, GEFs and GTPase Activating Proteins, GAPs (please see the cartoon, Figure Dii). In BC, RAC1 signaling has been demonstrated to involve enhanced upstream inputs of GEFs and reduced RAC inactivation by GAPs [31]. The role of Rho GTPases in BC has also been indicated from the report that progression of breast tumors is accompanied by a decrease in expression of the Rho guanine exchange factor TIAM1 and is inversely associated with several established breast tumor markers [68]. RAC activator, TIAM1 is a WP-responsive gene [69]. The fact that TIAM1 is one of the WP Target Genes has been reported in the “The Wnt Homepage” of 1997 - 2016 Roel Nusse (http://web.stanford.edu/group/nusselab/cgi-bin/lab/main; The Nusse Lab: Sandford University CA; Last updated: January 2016). Recently the phosphatidylinositol (3,4,5)-trisphosphate-dependent RAC Exchanger 1·Ras-related C3 botulinum toxin substrate 1 (P-Rex1·RAC1) complex revealed the basis of RAC1 activation in BC cells [70]. Engelman's group have identified P-Rex as a PIP3-dependent guanine exchange factor for RAC1 responsible for the activation of RAC1 in HER2 amplified and/or *PIK3CA* mutant BC cells [71]. We recently demonstrated that integrin-directed migration of BC cells can be abrogated by the inhibition of RAC1 activation following BEZ235 [72].

Since activation of PI3K pathway leads to blockade of cytosolic degradation of beta-catenin via GSK3beta (Figure 8B), it is plausible that an upregulated PI3K pathway (PTEN loss in the case of TNBC) may signal synergistically with WP in TNBC causing the activation of RAC1.

RAC1 was overexpressed or hyperactive in breast cancer tissues. The active variant RAC1b is found to be expressed in human breast tumors [65]. *How is RAC1 activated in breast cancers in the context of ID-MA phenotypes?* Emerging evidence supports a role for RAC-GEFs in the development and progression of breast cancer [31]. Also, there has been a correlation reported between TIAM1 expression and high tumor grade in human breast carcinomas [73]. MDA-MB231 expresses high levels of TIAM1 and is also know for its highly migratory and metastatic property [31]. The proto-oncogene VAV family, a RAC/Rho-GEF, is also known to be overexpressed in 81% of human breast tumors [31]. Thus, it is possible that beta1 integrin–mediated migratory/invasive phenotype involving F-actin is functionally associated to RAC1 via GEF in BC. VAV2 has been reported as an activator of both RAC1 and Cdc42 [74, 75]. Dhiraj Kumar et al., reviewed [76] that Wnt stimulated RAC1-GEF (VAV2) via Src-mediated tyrosine phosphorylation that further trigger RAC1 that regulate Nox1 activity and thus involved in Wnt-induced ROS production [77]. Hence we studied both TIAM1 and VAV2 mRNAs following Wnt pathway stimulation. MDA-MB231 cells developed spindle-shaped morphology mediated by cortical F-actin and occasional stress fibers during their movements in 3D collagen matrices very similar to fibroblasts utilizing beta1 integrin–mediated binding to collagen fibers for elongation and translocation which is further coupled to the cleavage and remodeling of ECM components using MMPs and other proteases [10]. Interestingly, MMP7 and MMP9 are transcriptional targets of WP. *How is RAC1 activation via GEF associated with cytosolic oncogenic signals including WP?* PREX2, VAV2 and TIAM1 are widely distributed GEF that activates the GTPase RAC1, is frequently mutated GEF in cancers. A recent study has identified that PTEN loss is dependent on p110beta via a p110beta-RAC1/2-positive-feedback loop in PTEN loss driven myeloid neoplasia [78]. In BC cell lines PTEN suppressed migration and invasion by blocking PREX2 activity, and PTEN inhibited the GEF activity of PREX2 toward RAC1 [79]. High basal Wnt activity in some mammary cancer cell lines has been reported to be associated with certain genes including the absence of PTEN [80]. Interestingly we have previously reported that a differential activation of WP increases MMP7 in a PTEN dependent manner in TNBC [46]. In our study, PTEN-null TNBC cell lines MDA-MB468, and SUM149 showed inhibition of fibronectin-induced RAC1 activity following WntC59, XAV939, sulindac sulfide and beta-catenin siRNA (Figure 3A, Figure 5E, Figure 6E). It can be speculated that the characteristic absence of PTEN protein in TNBC due to the loss of tumor suppressor gene PTEN, the most common first event associated with basal-like subtype [47], may be functionally connected to the activation of RAC1. However, it needs an in-depth study for a reasonable conclusion which is beyond the scope of this study. In this study, we provide evidence for the engagement of RAC1 in WP-mediated signal for ID-MA phenotypes. However, the mechanism of RAC1 activation in TNBC cells upon WP activation remains to be studied. In this context, theWP component, Disheveled (Figure 8B) has been proposed as one of the candidates as an upstream regulator of RAC1 activity as the overexpression of Dishevelled-2, but not Dishevelled-1 upregulated RAC1 in a different context [81]. TNBC patients’ (cBioPortal) tumor expressed alterations of *TIAM1* and *VAV2* genes (Figure 9A). We tested the nuclear localization of beta-catenin by confocal microscopy in MDA-MB231 cells (Figure 9B). In order to test the effect of sulindac sulfide on the WP, TNBC cells were first stimulated by LWnt3A condition media and mRNA for two of the WP downstream target genes of beta-catenin, cyclinD1 and cMYC were quantified by qRT-PCR in MDA-MB231BR cells and BT20 in the presence and absence of sulindac sulfide. Having confirmed the functional state of WP in TNBC cells (Figure 9B-9C), we determined that the activation of RAC1 is mediated via the increase in the mRNA levels of its *bona fide* exchange factors, TIAM1 and VAV2. This data was further strengthened by the treatment with sulindac sulfide (Figure 9Di) as represented schematically in Figure 9Dii. We set out to prove that LWnt3A CM stimulation in TNBC cells cause a significant increase in *TIAM1*, and *VAV2* mRNAs (Figure 9D). This data is in agreement with Valls et al., who reported that “upon Wnt stimulation, RAC1 activation requires RAC1 and VAV2 binding to p120-catenin” [82]. In line with the above fact, our data that *VAV2* mRNA (as well as *TIAM1* mRNA) is increased following WP stimulation (Figure 9D) explains the higher rate of migration as a result of RAC1-GPTase activation (pull down) and alteration of the actin cytoskeleton in TNBC cells.

As the routine treatment of BC is becoming more and more guided by the molecular markers present in the subtype-specific cancers, it has become imperative to identify the contribution of major pathways in TNBC in the conspicuous absence of classical targets of ER and HER2. Considering the characteristic aggressive nature of TNBC disease, such a premise becomes more compelling. The absence of hormone and growth factor receptors leaves limited opportunity for targeted therapies in this subtype of breast cancer especially in the context of the metastatic setting. In early stage recurrence, the significant pathway associated genes upregulated were MMPs and genes associated with cancer cell adhesion/motility including CDC42, RAC1 while a significant upregulation of WP genes including Wnt4 and Wnt16 was noted during stage IIIc recurrence [83]. Here we report the WP signals in ID-MA phenotypes through activation of RAC1-GTP-ase in TNBC. We present evidence for the first time to prove that RAC1 GTP-ase signals WP-stimulated ID-MA tumor cell phenotypes, migration, and invasion in TNBC. To the best of our knowledge, this is the first report to provide mechanistic evidence proving the role of RAC1 and Cdc42 in WP-mediated ID-MA phenotypes in TNBC. The alterations (gain/amplification) of RAC1 gene in ER-ve BC in the cBioPortal and poor outcome (RFS) data for ER-ve breast cancer cohort suggests the clinical significance of our finding in two independent data sets (Figure 9). Our data demonstrate a critical role for WP in TNBC progression and identified patients that may benefit from WP-RAC1-targeted therapies. We report for the first time that RAC1-activation via beta-catenin-TIAM1/VAV2 cascade acts as a downstream signaling event of WP activation in TNBC in the regulation of fibronectin-directed MA tumor cell phenotypes. Together, these results indicate that activation of a WP-RAC1 module contributes tumorigenic progression and identify a context-dependent role of GTP-ase in TNBC in the metastatic setting.

## MATERIALS AND METHODS

### Data analyses using cbioportal

We studied alterations (amplification, deep deletion, missense mutation, mRNA upregulation, mRNA downregulation, protein upregulation and protein downregulation) in *RAC1*, *TIAM1* and *VAV2* genes in Breast Invasive Carcinoma (TCGA, 2012) case set and in Invasive Ductal Cancers (brca/tcga/pub2015) case set using c-BioPortal. The genomic studies selected were (1) missense mutations, (2) amplifications (3) deep deletion (4) putative copy-number alteration from GISTIC and (5) mRNA expression Z-scores (microarray) with Z-score thresholds ± 2.0. In Breast Invasive Carcinoma (TCGA, 2012) case set, we have selected (1) all tumor samples (825 patients/825 samples) and (2) PAM50 Basal subtype (81 patients/81 samples). In the Invasive Ductal Cancer (brca/tcga/pub2015) case set, we have selected ER+ve breast tumors (594 cases/patients) and compared the data with ER-ve breast tumors (174 cases/patients). In Invasive Ductal Cancer (brca/tcga/pub2015) case set also, we have selected (1) all tumor samples (1105 cases/patients), (2) Invasive Ductal Cancer, Luminal A (201 cases/patients), (3) Invasive Ductal Cancer, PAM50 Luminal B (122 cases/patients) (4) Invasive Ductal Cancer, PAM50 Her2-enriched (51 cases/patients) and (5) Invasive Ductal Cancer, PAM50 Basal-like (107 cases/patients). Advanced cancer genomic data visualization is obtained with the help of “The Onco Query Language (OQL)”. OncoPrints represents compact means of visualizing distinctive genomic alterations, including somatic mutations, copy number variations (CNV), and mRNA expression changes occurring across a set of cases. We used the Onco Query Language (OQL) to select and define genetic alterations for all the OncoPrint outputs on the cBioPortal for Cancer Genomics. Oncoprints (different levels of zoom) have been generated using cBioPortal. OncoPrints are used for visualizing gene set as well as pathway alterations across a set of cases, and for visually identifying trends, such as trends in mutual exclusivity or co-occurrence between gene pairs within a gene set. Tumor types (tumor data sets) were chosen in accordance with the publication guidelines (as updated on January 17^th^, 2014) of TCGA (tcga@mail.nih.gov). cBioPortal data is subjected to scheduled updates. We acknowledge the cBioPortal for Cancer Genomics site (http://cbioportal.org) which provides a web resource for exploring, visualizing, and analyzing multidimensional cancer genomics data.

### Prognostic value of *RAC1* in ER-ve samples

The kmplot.com website was used to determine if RAC1 was prognostic of ER-ve samples in the Hungarian Academy of Science breast cancer cohort (http://www.ncbi.nlm.nih.gov/pubmed/20020197). The Affymetrix probe 208640_at for RAC1 was selected as the optimal JetSet probe where recurrence-free survival (RFS) represented the maximum number of samples. The user interface was used to select IHC determined ER-ve samples (n=671) where the Kaplan-Meier figure and log-rank analysis split on the median were automatically generated by kmplot.com. The user interface and meta-data associated with the combined independent cohorts did not allow for selection of a triple negative cohort with enough samples for survival analysis.

### Cell lines, reagents, drugs, RAC1 inhibitors and antibodies: cell lines, reagents drugs and antibodies

TNBC cell lines (HCC1937, MDA-MB231, MDA-MB468, BT20, Hs578t, and SUM149) were cultured according to a standard protocol as stated previously [84]. Our study included TNBC cell lines representing all major types of genetic alterations as described in detail in the discussion section. We used PTEN-null (MDA-MB468), CDKN2A-alterations (BT20, MDA-MB231), RAS/RAF mutated (MDA-MB231), RB1-alteration (MDA-MB468), BRCA1-competent (MDA-MB231, MDA-MB468), BRCA1-incompetent (HCC1937, SUM149), EGFR-overexpressed (MDA-MB468), p53-mutated (BT20, HCC1937, MDA-MB231, MDA-MB468) and PIK3CA-mutated (BT20) as well as histology like IDC (SUM149) and adenocarcinoma (MDA-MB231, MDA-MB468) in our study. TNBC cell line BT20 which expresses the WNT3 and the WNT7B oncogenes were used [85]. To test a direct involvement of WP in metastasis in TNBC, we used EGFP-tagged brain metastasis-specific cell line, MDA-MB231BR (a kind gift from Dr. P. S. Steeg; Women's Malignancies Branch, Center for Cancer Research, National Cancer Institute, NIH, Bethesda, MD, USA). All BC cell lines except SUM149 cell line were obtained from ATCC. The SUM149 cell line was obtained from Asterand (Partners in human tissue research). Antibodies including tubulin (BD Biosciences, CA), actin, active beta-catenin and beta-catenin (Abcam Inc., Cambridge, MA) were used for the study. Sulindac sulfide was obtained from Sigma-Aldrich. RAC1 inhibitors, NSC 23766 and W56 were procured from Tocris Bioscience (R&D system). NSC23766 is a selective inhibitor of RAC1-GEF interaction. NSC23766 prevents RAC1 activation by RAC-specific guanine nucleotide exchange factors (e.g. TIAM1) [86]. This compound was identified by a structure-based virtual screening of compounds that fit into a surface groove of RAC1 known to be critical for GEF specification [57].

On the other hand, RAC1 Inhibitor W56 is a peptide mimicking Trp (56) action interfering specifically by targeting RAC1 activation. This region between amino acids 53-72 of RAC1 is required for specific recognition and activation by the GEFs, and Trp (56) in beta (3) appears to be the critical determinant [87]. Since NSC23766, being a compound has been reported to also affect M2 mAchR and GPCR [86], we have used another more specific peptide inhibitor of RAC1, W56 in our study. RAC1 and Cdc42 pull down assay kit was obtained from EMD Millipore. Wnt3Arecombinant protein (Recombinant Human Wnt-3a Protein) was purchased from R&D systems. Recombinant Human Wnt-3a protein was reconstituted at 200 μg/mL in sterile PBS containing at least 0.1% human or bovine serum albumin. LWnt3A cell lines and the parental cell line were procured from ATCC. DAPI and Phalloidin-555 were procured from molecular probes. Fibronectin was procured from BD Biosciences. Beta-catenin siRNA and Lipofectamine 2000 were obtained from Invitrogen Inc. Transwell was obtained from Cornning Inc. WP modulators, WntC59 and XAV939, were procured from Cellagen Technology.

### Biochemical analysis

We performed immunoblots by Western blots on the equivalent amounts of protein (Bradford assay) using clarified cell lysates as mentioned earlier [88]. Cell lysates were assayed for total protein using BSA as standard. Normalized clarified lysates (25-50 μg protein) were resolved by 10% SDS-PAGE. Membranes were immunoblotted with different primary antibodies before individual bands on nitrocellulose membranes were visualized by ECL (Amersham Pharmacia Biotech, UK) using UVP.

### Quantification of mRNA by qRT-PCR

MDA-MB231BR and BT20 cells were plated in 6-well plates and stimulated with LWnt3A condition media (LWnt3A CM) in the absence and the presence of sulindac sulfide (25 μM and 50 μM). RNA was isolated from each sample using manufacturer's instructions (including on-column DNA digestion) via Qiagen RNEasy Mini kit (Cat 74104). RNA was analyzed for quality on a BioTek Synergy H1 microplate reader. The pure RNA (250 ng of per sample) was used to convert to cDNA via manufacture's instruction for Bio-Rad iScript Reverse Transcription Supermix for RT-qPCR kit (Cat 1708841). cDNA samples were diluted 1:20 and used with FastStart Essential DNA Green Master Mix (Cat 06402712001) along with custom primers for each gene of interest and analyzed on a Roche LightCycler 96 RT-qPCR instrument per manufacturer's instructions. LightCycler 96 software was used to produce melting analysis and relative expression data. Primers listed below are 5’-3’. GAPDH-F: GCACAAGAGGAAGAGAGAGACC, GAPDH-R: AGGGGAGATTCAGTGTGGTG, TIAM1-F: AAAG GCTGTGCATTCAATCCTG, TIAM1-R: TCAGTGC ACACAATCTTTTGCC, VAV2-F: TCGATGTGCGA GACTTTGGA, VAV2-R: CTTTGTTCTGCGCGATGCT, CCND1-F: CCGTCCATGCGGAAGATC, CCND1-R: GAAGACCTCCTCCTCGCACT, cMYC-F: AAACACA AACTTGAACAGCTAC, cMYC-R: ATTTGAGGC AGTTTACATTATGG. Cycling conditions varied per gene target.

### RAC1 and Cdc42 pull-down assay

GST-fusion protein pull-down assay was carried out using GST-fusion proteins, corresponding to the human PAK1 p21 binding domain (PBD, residues 67-150) expressed in E. coli. The final protein products were bound to glutathione agarose in liquid suspension, 300 μg of PAK1-PBD in 20 mM PBS, pH7.4, containing 50% glycerol. For the assay, 70-80% confluent cells were used for integrin engagement in the presence or absence of drugs on fibronectin-coated plates. Stimulated cells were lysed with extraction buffer (25 mM HEPES pH 7.5, 150 mM NaCl, 1% Igepol CA630, 10 MgCl2, 1 mM EDTA, 10% glycerol, 10 μg/ml leupeptin, 10 μg/ml aprotinin, 1 mM NaF, and 1 mM Na-orthovanadate). Following centrifugation, 10 μl PAK1 PBD (1 μg/ml) was added per lysate sample for 45 minutes at 4°C with gentle rocking. Agarose beads were collected (by pulse centrifugation for 10 seconds at 14,000 rpm), washed in extraction buffer, and resuspended in 30 μl Laemmli sample buffer to resolve protein by 15% SDS-PAGE. The membrane was probed with monoclonal anti-RAC1 and polyclonal anti-Cdc42 antibodies.

### Transient transfection of beta-catenin siRNA

Silencing of beta-catenin in TNBC cells was carried out using siRNA. Cell lines were transiently transfected with human-specific beta-catenin siRNA (Invitrogen Inc.) using Lipofectamine 2000 (Invitrogen Inc.) as described earlier [46]. In brief, cells grown in 6 well tissue culture plates were harvested following 72 hours of transfection with siRNA using Lipofectamine. Beta-catenin and active beta-catenin levels were determined by Western Blot.

### Integrin-directed migration and matrigel invasion Assays

Haptotaxis and wound healing assays were performed to test fibronectin-directed migration of tumor cells. Haptotaxis and matrigel invasion were carried out using transwell migration chambers. An adhesion assay on fibronectin was performed simultaneously with the haptotaxis assay under similar conditions. *In vitro* wound-healing migration assays were performed (scratch assay) as previously described by our group [45] [84] [88].

### Immunofluorescence staining of migrated cells in transwell migration following transient transfection of beta-catenin siRNA

The migration on fibronectin was tested by transwell assay. Cells transiently transfected with beta-catenin siRNA for 24 hours were used for the migration for 24 hours. The cells which did not migrate were removed from the upper side of the wells. Membranes of transwells were cut after 24 hours of migration and processed for Phalloidin-555 and DAPI staining to identify the migrated cells only.

### Actin dynamics and podia-parameters by confocal microscopy

TNBC cells were plated on fibronectin and treated with sulindac sulfide under conditions similar to that of the migration experiments. Cells were processed for Phalloidin-555 staining for filamentous actin. The nuclear localization of beta-catenin in the presence and absence of LWnt3A CM stimulation was stained with immunofluorescence (GFP) in MDA-MB231 cells. Nuclei were counterstained with DAPI. Cells were photographed using a Zeiss (Thornwood, NY) LSM 510 Meta confocal microscope with a 63 x (1.4-numerical-aperture) or 40 x (1.4-numerical-aperture) Plan-Apochromat oil objective as mentioned earlier [84]. Lamellipodia/cell and filopodia/cell were identified under a confocal microscope. For the semi-quantification purpose, an equal number of cells from the control and treated experiments exhibiting, at least, one filopodia were chosen from randomly selected fields, and their screenshot images were collected. A total number of lamellipodia/cell and filopodia/cell from the control, as well as treated groups, were counted manually from these screenshot confocal images.

### LWnt3A conditioned media and Wnt3A recombinant protein

LWnt3A conditioned medium was obtained from the LWnt-3A (ATCC^®^ CRL-2647™) cells [50]. These cells L-M(TK-) cells which were transfected with a Wnt-3A expression vector and stable clones were selected in medium containing G418. The *Wnt3A* gene encodes a secreted glycoprotein. The cells are engineered to secrete biologically active Wnt3A protein. We used these cells as our source for production of Wnt3A conditioned medium (Wnt3ACM) (as described by the ATCC). L Cells (ATCC® CRL2648™) was used as the parental line for the LWnt3A cell line. Wnt3A recombinant protein was diluted as suggested by the manufacturer.

### Statistical analysis

Biochemical experiments were independently repeated 3-4 times. Phenotypic experiments were performed in quadruplets. The Student's t-test was used to evaluate differences between treated and vehicle controls. Data presented in the graphs represent the Mean ± S.D. of results. The minimum level of statistical significance was set at p<0.05. Inter-group comparison was made with a paired two-tailed (Student's t-test).

## SUPPLEMENTARY MATERIAL FIGURE


