# Supplementary material for: RAC1 GTP-ase signals Wnt-beta-catenin pathway mediated integrin-directed metastasis-associated tumor cell phenotypes in triple negative breast cancers
Source: Oncotarget. 2016 Nov 25;8(2):3072–103. doi: 10.18632/oncotarget.13618 (PMC5356866; doi:10.18632/oncotarget.13618)
Supplement: Supplementary file 1 [file oncotarget-08-3072-s001.pdf]

RAC1 GTP-ase signals Wnt-beta-catenin pathway mediated integrin-directed metastasis-associated tumor cell phenotypes in triple negative breast cancers

SUPPLEMENTARY FIGURE

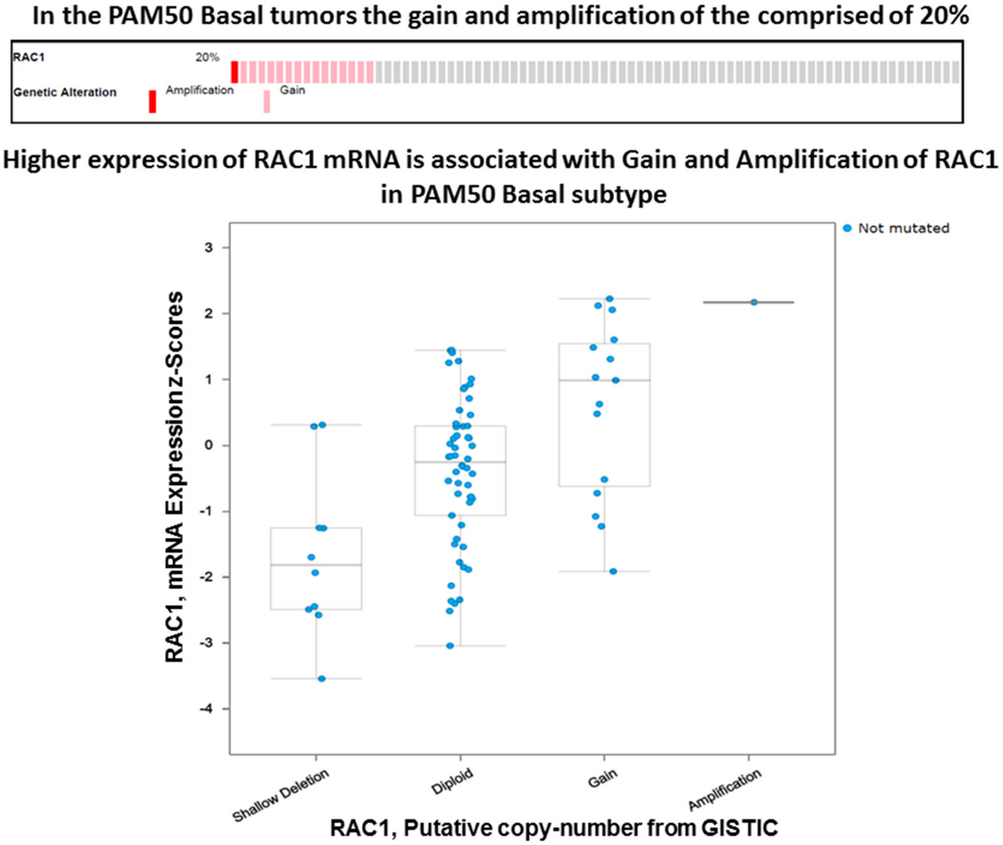

Supplementary Figure S1: Gain and amplification of *RAC1* gene in PAM50 basal tumors comprises of 20% of the total alterations (upper oncoprint). Higher expression of RAC1 mRNA is associated with Gain and Amplification of RAC1 in PAM50 Basal subtype (lower panel). RAC1 mRNA expression is plotted against RAC1 putative copy-number from GISTIC as obtained from cBioPortal.
